# Multilevel instrumented posterolateral lumbar spine fusion with an allogeneic cellular bone graft

**DOI:** 10.1186/s13018-019-1424-2

**Published:** 2019-11-15

**Authors:** John F. Hall, Julie B. McLean, Savannah M. Jones, Mark A. Moore, Michelle D. Nicholson, Kimberly A. Dorsch

**Affiliations:** 1 Flagstaff Bone and Joint, Flagstaff, AZ USA; 2 LifeNet Health, 1864 Concert Drive, Virginia Beach, VA 23455 USA

**Keywords:** Posterolateral fusion, Low back pain, Multilevel, Cellular bone allograft, Bone regeneration, Allograft

## Abstract

**Background:**

Low back pain (LBP) is the leading cause of absence from work, disability, and impaired quality of life. Fusion surgery may be indicated when non-operative treatments have failed to provide relief. Surgery may include the use of fusion-enhancing implants, such as cellular bone allografts (CBAs). The purpose of this retrospective study was to evaluate efficacy and safety of one CBA (V-CBA) in patients who underwent instrumented posterolateral fusion (IPLF).

**Methods:**

Retrospective data were collected from 150 consecutive patients who had undergone IPLF surgery between January 1, 2015, and March 31, 2018, in which V-CBA was used. All surgeries were performed by one surgeon. V-CBA was mixed with local autograft bone. Patient diagnoses included degenerative disc disease, spondylosis, spondylolisthesis, or spondylolysis with or without stenosis. Standing anteroposterior (AP) and lateral images were collected prior to surgery and again at the terminal visit, which took place between 6 and 33 months post-operatively. De-identified images were assessed radiologically. Adverse events were documented. The primary composite endpoint of fusion status was dependent upon two main criteria: bridging bone per the Lenke scale (classified as “A” definitely solid or “B” possibly solid) and posterior hardware status (intact). Lenke scale C or D were categorized as pseudarthrosis.

**Results:**

Eighty-seven male and 63 female patients (613 levels total) underwent IPLF in which V-CBA was implanted. An average of 4.1 levels was treated, with 59.3% of patients having undergone treatment for more than 3 levels. Twenty-nine percent of patients had diabetes. Fifty-two percent of patients had previously used nicotine products, and 12% were current smokers. Sixteen serious adverse events were recorded and included lumbar seroma, cerebrospinal fluid leak, wound dehiscence, pneumonia, urinary tract infection, and myocardial infarction. Successful fusion (Lenke scale “A” or “B”) was recorded in 148 out of 150 patients (98.7%), or 608 out of 613 levels. The total pseudarthrosis rate was 0.8%.

**Conclusions:**

The use of V-CBA combined with local autograft in multilevel IPLF resulted in successful fusions in 98.7% of patients. These results are particularly robust given the complex nature of many of these cases: 89 patients had 4 or more surgical levels, and many patients had multiple comorbidities.

**Level of evidence:**

IV

## Introduction

Worldwide, low back pain (LBP) is the leading cause of absence from work, disability, and impaired quality of life [1]. Conservative treatment options may include physical therapy, epidural steroid injections, nonsteroidal anti-inflammatory drugs (NSAIDs), analgesic medications, neuromodulatory drugs, spinal manipulation, acupuncture, biofeedback, and nerve block therapies. If these treatments fail to relieve pain or other symptoms, surgery may become necessary. Spinal fusion surgery is one of the most commonly performed surgeries in the world and is indicated when the patient has exhausted all appropriate non-operative treatments and has one or more of the following: a significant component of axial pain, spinal deformity, instability, or a situation in which adequate decompression for radicular symptoms cannot be achieved without causing iatrogenic instability [2]. Fusion may be achieved using local or distant autograft (typically from the iliac crest), synthetics, or allograft.

Autograft bone, taken from an additional surgery site on the patient, is often used during fusion procedures. However, iliac crest bone graft (ICBG) is associated with significant donor site morbidity in up to 30% of patients, an increase in operative time, blood loss, and risks of infection, cosmetic deformity, and arterial or nerve injury [3]. Furthermore, the supply of available autologous ICBG is limited, and the quality of the autograft bone may be poor depending upon underlying factors. Alternatively, autograft local bone may be used to help achieve fusion. Although volume is limited, this bone is typically available at the surgical site of primary fusions, particularly when performing decompressions for stenosis in conjunction with the fusion surgery.

Numerous autograft alternatives have been developed with the intention of enhancing fusion success, and mostly fall into one of the following categories: synthetic ceramics, allografts, recombinant proteins, and xenografts. Cellular bone allografts (CBAs) are a newer alternative that typically contain viable mesenchymal stem cells (MSCs) as well as osteoconductive and osteoinductive bone components. Although MSCs have the potential to differentiate into bone cells, they may differentiate into unwanted cell types as well. Additionally, the differentiation process is time-consuming and complex, depending upon the local microenvironment which may lack the appropriate signals for osteogenesis in some patients. As such, recent studies provide evidence that bone cells such as osteoblasts and osteocytes may be preferred over mesenchymal stem cells for bone fusion [4–6]. In response, one type of CBA, ViviGen (V-CBA), was developed to contain viable, lineage-committed bone cells in an osteoconductive corticocancellous matrix. It also includes demineralized bone with osteoinductive potential, thereby providing all vital elements for the initiation of new bone growth while avoiding the uncertainty of MSC-based CBAs that may differentiate into undesirable cell types such as adipocytes. The purpose of this study was to evaluate the efficacy and safety of V-CBA in patients who had undergone instrumented posterolateral fusion (IPLF) without interbody cages.

## Methods

Approval was received from the Western Institutional Review Board (IRB) to conduct a retrospective review of data collected from all IPLF cases performed between January 1, 2015, and March 31, 2018, in which V-CBA (ViviGen®, LifeNet Health, Virginia Beach, VA, USA) was used to treat patients who had failed non-operative treatment, continued to have symptomatic back and/or leg pain, instability or deformity, and had radiographic evidence consistent with these symptoms. To be considered eligible for data collection, patients must have met all inclusion criteria, including having been over age 21 at the time of surgery and having had an indication for spinal fusion surgery: degenerative disc disease, spondylosis, spondylolisthesis, or spondylolysis with or without stenosis. Diagnosis of degenerative disc disease required back and/or leg (radicular) pain along with instability (≥3 mm translation or ≥ 5^o^ angulation), or MRI confirmation of Modic Type 1 or Type 2 changes, or high-intensity zones in the disc space. Patients must not have met any exclusion criteria, including the use of a synthetic (e.g., PEEK) interbody spacer within the procedure and also having had less than 6 months of follow-up.

The primary endpoint was a successful fusion rate assessed with anteroposterior radiographs of the lumbar spine. Secondary endpoints included operating room procedure time, blood loss, post-operative patient self-assessments to evaluate back and leg pain on a visual analog scale (VAS), and patient satisfaction.

### Surgical technique

All study patients underwent IPLF with bone grafting. Instruments used were Expedium® and Expedium Verse® Spine Systems (DePuy Synthes, Raynham, MA, USA). The fusion procedures were performed by the lead investigator. The resulting local bone and V-CBA were mixed together and implanted on each side of the spine. Patients were given the opportunity to have blood returned by cell saver during the procedure. All patients had prophylactic antibiotic therapy during the perioperative period.

### Assessment methods

Standing AP and lateral images were collected prior to surgery and at the terminal visit. To provide a definitive assessment of fusion integrity, a radiological assessment of all available imaging was performed. Images were de-identified prior to the fusion assessment to assure appropriate blinding. The primary composite endpoint of fusion status was dependent upon two main criteria: bridging bone per the Lenke scale [7] (classified as possibly solid or definitely solid) and posterior hardware status (intact). The area was judged as fused if there was definitive, uninterrupted bridging of well-mineralized bone between the transverse processes lateral to instrumentation, with bony continuity and trabeculation indicating bone maturation. Successful fusion required all components to be met at all levels of surgery.

### Statistical methods

All statistical analyses were calculated using Stata (StataCorp, College Station, TX, USA) and Prism version 7.00 for Windows (GraphPad Software, La Jolla, CA, USA). Continuous data were summarized by descriptive statistics of *n*, mean, median, standard deviation, minimum, and maximum. Categorical data were summarized by frequencies and percentages. Safety data were summarized with descriptive statistics.

Safety was assessed by the incidence of treatment-associated adverse events (AEs) including any complications during surgery, neurological worsening from baseline, or need for revision. Intraoperative metrics included operative time, blood loss, and number of levels treated. Operative time was defined as the total time the patient was in the operating room (OR), from entry to exit, including anesthesia induction, surgery preparation time, and extubation.

## Results

Data were collected from 150 consecutive patients (87 male and 63 female) undergoing IPLF in which V-CBA was implanted (Table 1). The median age of patients at the time of surgery was 70 ± 9 years (males, 69 ± 10, females, 70 ± 9) with a median BMI of 31 ± 6. Twenty-nine percent of patients had a diagnosis of diabetes, while 13% had a prior diagnosis of cancer. The most common diagnoses were instability, stenosis, frontal and sagittal plane deformity, spondylolisthesis, and radiculopathy. Back pain with bilateral lower extremity radiculopathy was present in 74.7% of patients while 13% and 10% experienced right or left radiculopathy, respectively. Two percent had generalized back pain.

**Table 1:** Patient demographics

Participants (*n*)	150
Age (years)^a^	70 ± 9
Sex	
Male	87 (58%)
Female	63 (42%)
BMI (kg/m^2^) ^a^	31 ± 6
Diabetes	
Yes	43 (29%)
No	107 (71%)
History of tobacco use	
Yes	78 (52%)
No	72 (48%)
Current nicotine use	
Yes	18 (12%)
No	132 (88%)
Prior cancer diagnosis	
Yes	20 (13%)
No	130 (87%)
Previous spinal fusion	
Yes	14 (9%)
No	136 (91%)

^a^Continuous data is expressed as mean ± SD unless indicated otherwise

Thirty-four percent (30/87) of the males and 21% (13/63) of the females were being treated for diabetes with a stable regimen of antidiabetic medications (90% of diabetic males, 92% of diabetic females) or insulin (27% of males, 8% of females).

Twelve males and 8 females had a prior history of cancer. Males reported previous diagnoses and treatments for skin, prostate, kidney, throat, and esophageal cancers. Female patients reported leukemia, breast, skin, and kidney cancers.

Fifty-two percent of patients had previously used nicotine products and 12% were current smokers.

Prior to this study, patients used or were prescribed one or more non-surgical or surgical treatments; not all pre-operative treatments were prescribed by the principal investigator (Table 2). Physical therapy and epidural spinal injections were prescribed for 96% and 84.7% of patients, respectively. Exercise for pain relief was used by 67.3% of patients while 31.3% tried stretching. No patients sought cognitive behavior therapy and few used acupuncture, braces, or chiropractic interventions. Over half of the patients (58.7%) used non-steroidal anti-inflammatory drugs. Forty-two percent of patients were taking a narcotic prior to surgery, while 16.7% used medications approved for neuropathy and 15.3% used muscle relaxers. Twenty-six of the patients (17.3%) had a prior spinal fusion procedure at levels not described in the current study: 16 cervical, 6 lumbar, and 4 thoracic.

**Table 2:** Treatments prescribed or used prior to surgery

Physical therapy	144 (96%)
Epidural spinal injections	127 (85%)
Exercise	101 (67%)
Stretching	47 (31%)
Hot/cold therapy	23 (15%)
Weight loss	17 (11%)
Chiropractor	15 (10%)
Prior lumbar surgery	14 (9%)
Facet injections	4 (3%)
Brace	2 (1%)
Acupuncture	2 (1%)
None	2 (1%)
Diagnostic injections	1 (< 1%)
Pain management	1 (< 1%)

**Fig. 1:**
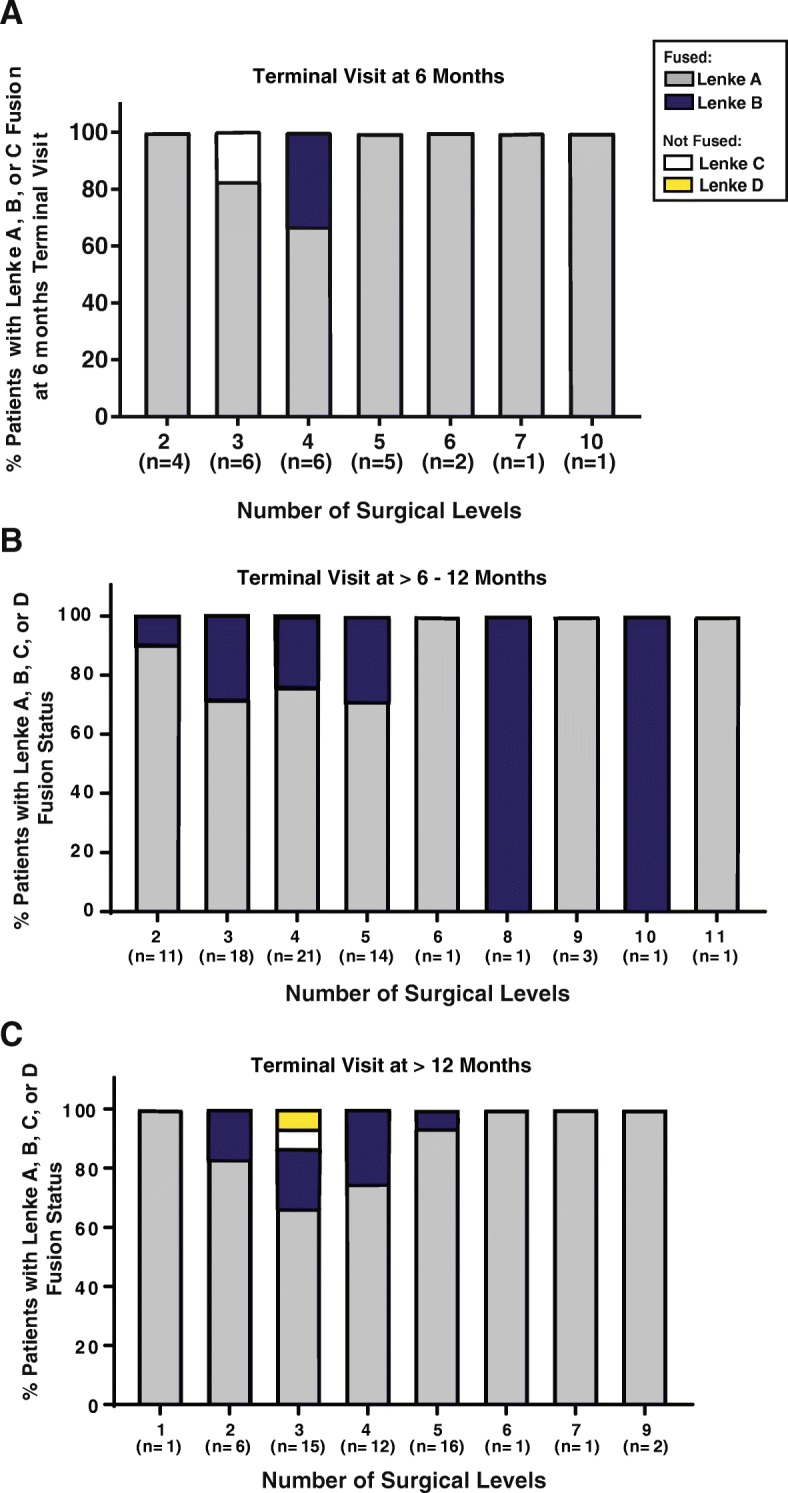
Fusion status as terminal visit. Terminal visits were divided into post-operative time periods: at 6 months, > 6–12 months, and > 12 months. The *X*-axis describes the number of patients at each surgical level in the respective terminal visit time period. The *Y*-axis describes the percent of patients at each surgical level for each terminal time period that had Lenke fusion A, B, C, or D. **a** For the terminal visits at 6 months, 96% of patients were considered fused (80 Lenke A and 16% Lenke B), while 4% were not fused (Lenke C). **b** For patients who had post-operative terminal visits > 6–12 months, 100% were considered fused (76% Lenke A and 24% Lenke B). **c** For the patients whose terminal visit happened after 12 months post-operatively, 98% were considered fused (83% Lenke A, 15% Lenke B, 2% Lenke D)

**Fig. 2:**
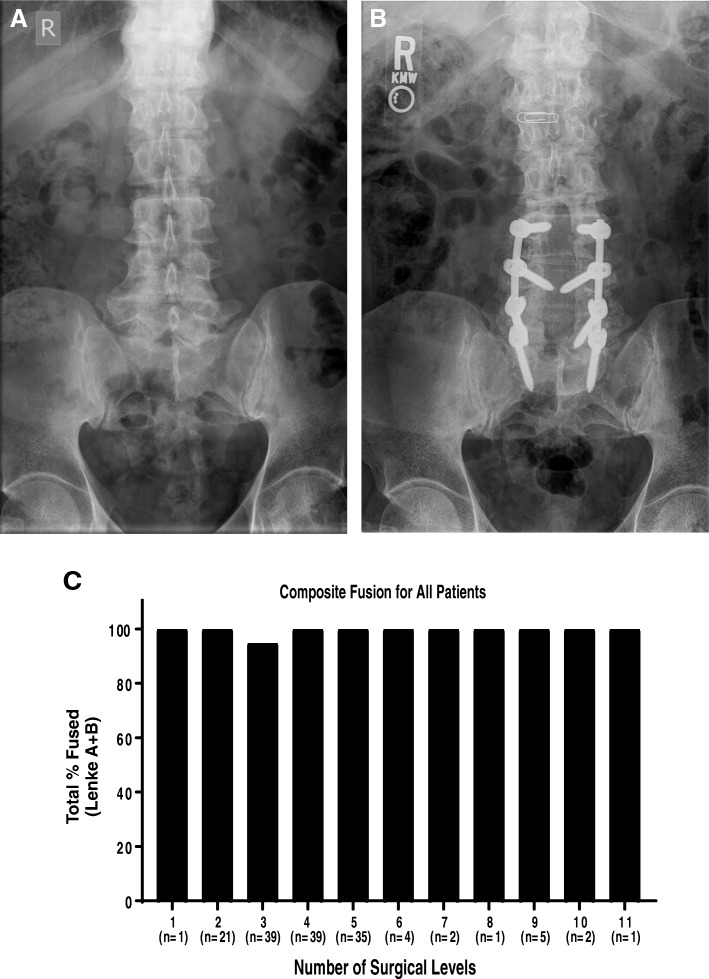
Representative radiographs and combined fusion for all patients. **a** 69-year-old male, former smoker with a body mass index of 26 presented with back pain and left lower extremity radicular pain. Conservative treatment included physical therapy, stretching, epidural spinal injections, and NSAIDs. **b** Seven months after L4-S1 decompression and fusion were performed, the patient was fused (Lenke A). **c** Combined fusion graph (Lenke A + Lenke B) shows that 148 of 150 patients (98.7%) were considered fused: 119 patients Lenke A and 29 Lenke B

**Fig. 3:**
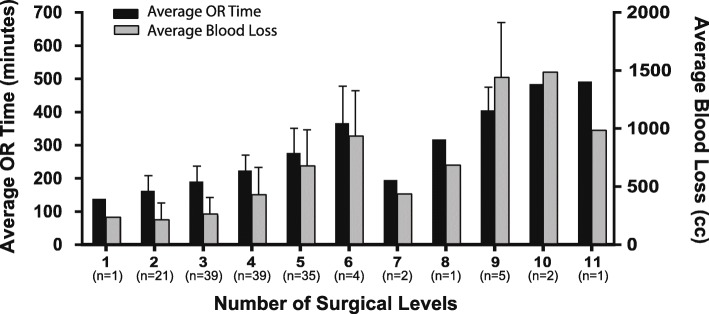
The relationship between the number of surgical levels, operating room (OR) time, and blood loss. As the number of surgical levels increases, the amount of time spent in the OR (minutes) and blood loss (cc) generally increased (mean/SD)

V-CBA was used to treat 613 levels in 150 patients. An average of 4.1 levels was treated via IPLF surgery, with 59.3% of patients undergoing treatment for more than 3 levels. Fusion status was assessed at each patient’s terminal visit, which occurred from 6 to 33 months postoperatively. Due to the variability in follow-up time, fusion status in relation to the number of surgical levels was broken into 3 different time periods: patients with final appointments at 6 months, greater than 6 months to 12 months, and more than 12 months (Fig. 1a–c). All images for each patient were blinded and, using the Lenke classification system, the surgeon reviewed the images and assigned a grade for the fusion. See Fig. 2a, b for representative images. Successful fusion was defined as either A (definitely solid fusion with bilateral stout fusion masses present) or B (possible solid with unilateral large fusion mass and a contralateral small fusion mass). Pseudarthrosis was defined as a grade of C (probably not solid with a fusion mass bilaterally) or D (definitely not solid with bone graft resorption or obvious pseudarthrosis), even if one was not recorded in the clinical notes. Successful fusion (score of A or B) was recorded in 148 out of 150 patients (98.7%, Fig. 2c). The total pseudarthrosis rate was 5 out of 613 PLF levels (0.8%). One diabetic, morbidly obese (BMI = 42) male patient in the 3 levels group returned 6 months post-procedure and was assessed as a grade C. Nonetheless, he reported complete resolution of back and radicular left limb pain (VAS = 0) and was very satisfied with the results. The other patient assessed as not fused was given a D at the final clinic visit 13 months post-procedure. The female L4-S1 fusion patient reported a fall at 2 months post-procedure, fracturing the left S1 pedicle and developing L5-S1 pseudarthrosis, which required subsequent surgical repair. The assessor noted that L5-S1 was not fused although L4-L5 did have solid fusion at that visit. The patient reported pain (VAS = 6) at the final visit and was unsatisfied with the results. It was also noted that as the number of surgical levels increased, OR time (OR entry to exit) and average blood loss also increased (Fig. 3). Average VAS and patient satisfaction ratings at terminal visit are reported in Table 3.

**Table 3:** Average VAS (1–10) and patient satisfaction per number of surgical levels

Number of levels	Average VAS^a^	Average patient satisfaction
1	0.00	Very satisfied
2	1.62	Satisfied to very satisfied
3	2.15	Satisfied to very satisfied
4	2.05	Satisfied to very satisfied
5	1.97	Satisfied to very satisfied
6	3.25	Satisfied to very satisfied
7	1.50	Very satisfied
8	2.00	Satisfied to very satisfied
9	1.20	Satisfied to very satisfied
10	1.00	Very satisfied
11	4.00	Satisfied to very satisfied

^a^Scale from 1 to 10, where 10 is the worst pain

Fusion data were further analyzed for the effect of diabetes, age, and current smoking status. Forty-two of 43 (97.7%) patients with diabetes had successful fusion. The single diabetic patient who failed to fuse was 73 years old. The other patient, a female without a history of diabetes, who failed to fuse at L5-S1 was 68 years old. All patients who were current smokers fused. Gender, previous spine surgery at the index level, and the total number of surgical levels did not affect the overall fusion rates.

In addition to the patient who had a failure due to a fall, a second patient also fell 2 months after surgery and subsequently sought treatment for severe low back pain. A pseudarthrosis of L5-S1 required an anterior lumbar interbody fusion procedure with the removal of L5-S1 hardware. One patient was involved in a motor vehicle accident 9 months after surgery and presented with increased right-sided paraspinal lumbar pain without radiculopathy. The pain resolved after treatment with NSAIDs and physical therapy. Another patient had a broken L5 screw about 2 weeks after IPLF. A surgical reintervention without additional V-CBA was performed to replace the hardware. Eleven months post -IPLF, one patient required a reintervention for recurrent stenosis of L5-S1. A foraminotomy was performed which resolved the radicular pain. Finally, approximately 9 months after IPLF, an L3-S1 fusion patient reported increasing low back pain. L2–3 proximal junctional kyphosis was diagnosed. A surgical reintervention using V-CBA was performed to extend the fusion levels to L2.

A total of 16 serious adverse events (SAEs) were documented in 15 patients. Despite SAEs, all had achieved successful fusions when assessed at their final visits. Ten patients had events resulting in a longer hospital length of stay. Five patients (1 at 4 surgical levels; 4 at 5 surgical levels), two of those with diabetes, developed lumbar seromas shortly after surgery which were promptly evacuated during the hospitalization. One diabetic, morbidly obese (BMI = 41) patient (2 surgical levels) had a cerebrospinal fluid leak and required repair of a durotomy. Two patients, both with nine surgical levels, one a diabetic, developed dehisced wounds that required surgical closure. One morbidly obese (BMI = 43) patient with a 10-level fusion and a history of chronic obstructive pulmonary disease (COPD) and urinary retention was diagnosed with pneumonia and a urinary tract infection after surgery. Another patient (four surgical levels) with a history of hypercholesterolemia, hypertension, COPD, and who was also a current smoker was diagnosed with myocardial infarction after the surgical procedure.

## Discussion

The results of this retrospective study demonstrate that IPLF with V-CBA and local bone results in excellent fusion rates across multiple surgical levels with few complications. After an average of 12 months, 98.7% of patients had successful fusion.

Although ICBG has been used successfully for decades in spinal fusion procedures, there is a concern regarding morbidity associated with the harvesting procedure. As an alternative, surgeons have studied the use of local autologous laminectomy bone alone in IPLF. Ohtori et al. randomized patients undergoing single-level posterior lumbar decompression and fusion to receive either local bone or iliac crest autograft and compared fusion rates at 2 years. The authors found no statistical differences between the two groups, both of which resulted in fusion rates of 90% [8]. Lee et al. also presented a case series of 182 patients who had undergone single-level PLF with local bone alone, which resulted in a 93% fusion rate at the end of the follow-up period of at least 18 months [9]. Finally, Sengupta et al. presented a series of 112 consecutive patients with either iliac crest or local bone in 1- to 4-level IPLF. The iliac crest group had higher fusion rates (75%) compared to local bone alone (65%) [10]. Although these local bone-only fusion results were acceptable, the limited quantity of local bone available can be problematic, particularly in multilevel fusions. Additionally, in the Sengupta et al. study, the fusion rate declined as the number of surgical levels increased. Another concern is that local bone collected during a laminectomy is predominantly cortical bone. Compared to cancellous bone, cortical bone has fewer lineage-committed bone cells and is less biologically active. The concerns regarding limited quantity and activity of local bone have led to speculation on whether a bone graft substitute might be an efficacious alternative.

Cammisa et al. compared the effectiveness of Grafton® DBM gel (Medtronic, Minneapolis, MN) with iliac crest autograft in posterolateral fusion patients [11]. A total of 120 patients underwent surgery with pedicle screw fixation and bone grafting. Iliac crest autograft was implanted on one side of the spine and Grafton DBM gel was implanted on the contralateral side in the same patient. The bone graft mass was fused in 42 cases (52%) on the Grafton DBM side and in 44 cases (54%) on the autograft side. The reintervention rate for pseudarthrosis was 1.0% (1 out of 81 patients) in this study. As reported here, V-CBA provided higher fusion rates than either Grafton or autograft, by 46% and 44% respectively. It was also comparable for reintervention for pseudarthrosis [occurred in 3 of the 150 subjects in our study (2.0%)].

One of the most commonly used grafts to replace autograft is recombinant bone morphogenetic protein-2 (rhBMP-2). Glassman et al. studied the clinical, radiographic, and economic outcomes in 102 patients over the age of 60, who had undergone posterolateral fusion with either rhBMP-2 (Infuse™ bone graft, Medtronic, Minneapolis, MN, USA) or ICBG [12]. The mean number of surgical levels in the randomized, controlled trial was 1.96 for the rhBMP-2 group and 1.98 for the ICBG group. The mean operative time for the rhBMP group was 248 ± 58.5 min and 270 ± 33.6 for the ICBG group. At 24 months, the rhBMP-2 group had an 83.6% fusion rate while the ICBG group had a 70.8% fusion rate. Surgical reintervention was required in 8% of the rhBMP-2 group and in 21% of the ICBG group. In our study, the mean operative time for V-CBA was 211.1 ± 87.3 across all fusion levels, which is 37 min and 59 min faster than rhBMP-2 and ICBG, respectively. The fusion rate for the 21 patients that had 1 and 2 level fusions with V-CBA was 97.6%, compared to 83.6% for rhBMP-2 and 70.8% for ICBG. In addition, surgical reintervention was required in only 5 of the 150 V-CBA patients (3.3%).

Although the data presented here demonstrate robust fusion in 150 patients and are favorable compared to other grafting options, we recognize several limitations inherent in the study design. The data were collected from one hospital and represent the experience of one surgeon. The results may not be applicable to other study centers. The data were collected retrospectively, without a comparative control or competitive arm. The retrospective nature of the study did allow for calculation of fusion rates across multilevel procedures from radiographs using a standardized classification, listing of complications, and basic patient demographics with a medical history. Additionally, VAS pain scores were not charted prior to surgery and fusion was calculated at the last visit with the surgeon instead of at the industry standard of 12 or 24 months. Another limitation is the inability to differentiate the relative contributing factors of V-CBA and local bone since a combination was used in these surgeries. An average of 8.7 cc of V-CBA was used with 32.4 g of local bone across all study participants, with these values varying according to the number of surgical levels, quantity and quality of local bone, and patient comorbidities, particularly diabetes and tobacco use. However, insight can still be gained comparing the results presented here to the studies mentioned above, which suggest a much lower rate of fusion when only the local bone was used. Future studies should investigate the relationship between fusion status and the ratio of V-CBA to the local bone.

## Conclusion

This study reports the efficacy of using V-CBA in standalone IPLF. V-CBA includes all three vital elements—osteoconductivity, osteoinductivity, and osteogenecity—for the initiation of new bone growth. Fusion rates, 98.7%, were higher than those found in literature for iliac crest or local bone autograft alone as well as other grafting options. As demonstrated, V-CBA can be used in conjunction with the local bone to achieve solid multilevel fusion. It contains viable, lineage-committed bone cells in an osteoconductive, cortico-cancellous matrix. It also includes demineralized bone with osteoinductive potential, thereby providing all vital elements for the initiation of new bone growth while avoiding the uncertainty of MSC-based CBAs that may differentiate into unwanted cell types. The use of V-CBA combined with local autograft in IPLF resulted in successful fusions in 98.7% of patients (608/613 levels). These results are particularly robust given the complex nature of many of these cases: 89 patients had 4 or more surgical levels, and many patients had multiple comorbidities.

## Data Availability

All data generated or analyzed during this study are included in this published article.

## Abbreviations

CBA: Cellular bone allograft
DBM: Demineralized bone matrix
ICBG: Iliac crest bone graft
IPLF: Instrumented posterolateral fusion
LBP: Low back pain
MSCs: Mesenchymal stem cells
NSAIDs: Nonsteroidal anti-inflammatory drugs
rhBMP: Recombinant human bone morphogenetic proteins
V-CBA: ViviGen cellular bone allograft
